# Reply to: “A double bond with weak σ- and strong π-interactions is still a double bond”

**DOI:** 10.1038/s41467-021-24239-w

**Published:** 2021-06-29

**Authors:** Soichiro Kyushin, Yoshikuni Kurosaki, Kyohei Otsuka, Haruna Imai, Shintaro Ishida, Toru Kyomen, Minoru Hanaya, Hideyuki Matsumoto

**Affiliations:** 1 Division of Molecular Science, Graduate School of Science and Technology, Gunma University, Kiryu, Gunma, Japan; 2 Department of Chemistry, Graduate School of Science, Tohoku University, Sendai, Japan; 3 Division of Pure and Applied Science, Graduate School of Science and Technology, Gunma University, Maebashi, Gunma, Japan

**Keywords:** Chemical bonding, Organometallic chemistry

**Replying to** C. Foroutan-Nejad *Nature Communications* 10.1038/s41467-021-24238-x (2021)

**Fig. 1:**
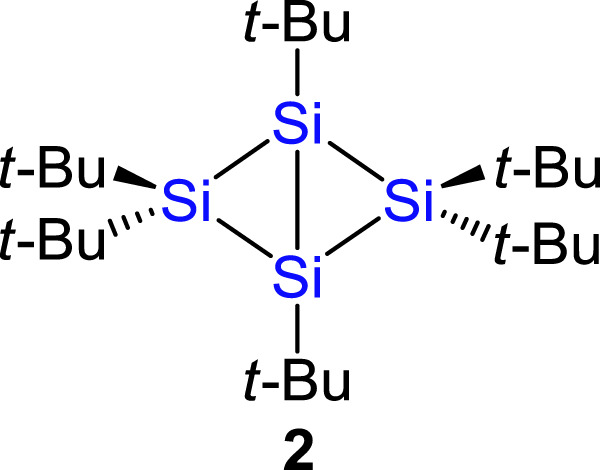
**Chemical structure of 2.**

In our original paper^1^, we reported synthesis of 1,2,2,3,4,4-hexa-*tert*-butylbicyclo[1.1.0]tetrasilane (**2**, Fig. 1). The X-ray crystallography of **2** showed a planar geometry around the bridgehead silicon atom (angle sums except for the inter-bridgehead bond = 359.79°). On the basis of experimental results of X-ray crystallography, electron paramagnetic resonance, magnetic susceptibility, UV/Vis and ^29^Si NMR spectra, and theoretical calculations including natural bond orbital analysis, we concluded that **2** has a silicon–silicon **π** single bond between the bridgehead silicon atoms. We have been aware that the HOMO–6 and HOMO–1 represent in-phase and out-of-phase orbital interactions between the linearly arranged two σ(Si_bridgehead_–C_*tert*-butyl_) orbitals, respectively. We did not discuss them in the original paper because (i) we considered that the σ-type bonding interaction between the bridgehead silicon atoms due to the in-phase interaction (HOMO–6) should be canceled out by the corresponding out-of-phase interaction (HOMO–1) and (ii) we did not find σ-type interaction between the bridgehead silicon atoms within the above-mentioned our theoretical investigation (output threshold > 2.1 kJ mol^–1^).

In their commentary, Dr. Foroutan-Nejad took a different approach to characterize **2**. The author analyzed electron density of **2** theoretically and concluded that the silicon–silicon π bond is accompanied by a weak but non-negligible **σ** bond. We welcome discussion about the unusual bonding situation in the isolable compound **2** from diverse viewpoints to obtain deeper understanding of this chemical bond.
